# Atopic dermatitis: Tofacitinib, an option for refractory disease

**DOI:** 10.1002/ccr3.3325

**Published:** 2020-10-11

**Authors:** Sineida Berbert Ferreira, Rachel Berbert Ferreira, Morton Aaron Scheinberg

**Affiliations:** ^1^ Private Clinic – Dermatologia Dra. Sineida Berbert Ferreira Maringa Brazil; ^2^ Faculdade de Medicina Centro Universitario Cesumar Maringa Brazil; ^3^ Hospital Israelita Albert Einstein Sao Paulo Sao Paulo Brazil

**Keywords:** atopic dermatitis, dermatology, eczema, JAK inhibitors, tofacitinib

## Abstract

Atopic dermatitis (AD) is a common skin disease, associated with high burden impact in quality of live, in moderate‐severe disease severity. Several targeted drugs are under development for AD. Here, we present a patient with refractory disease to systemic traditional immunosuppressive drugs, treated successfully with oral tofacitinib, with complete response.

## Graphical abstract

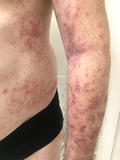

## 1 INTRODUCTION

Atopic dermatitis (AD) is an autoimmune inflammatory disease, characterized by xerosis, eczematous lesions, redness, persistent, and severe pruritus. In this case report, we show a different option of treatment for severe, chronic refractory cases.

## 2 CASE REPORT

**FIGURE 1:**
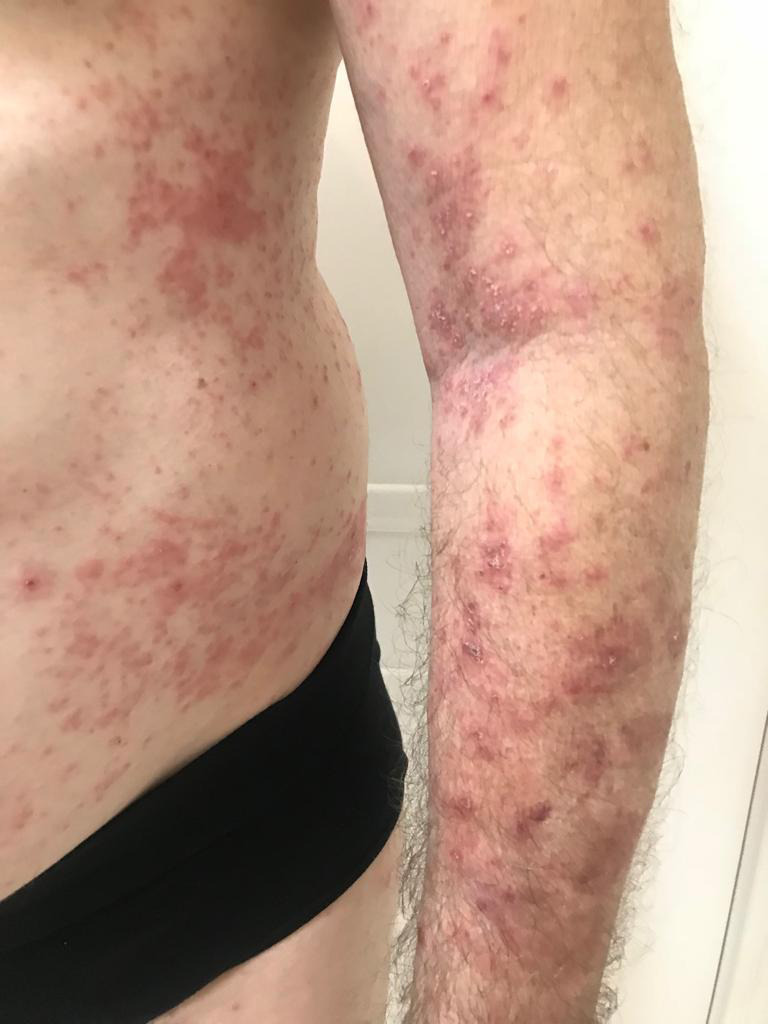
Before treatment with tofacitinib 5 mg/BID; previous treatments included topical corticosteroids, tacrolimus ointment, moisturizers, prednisone, methotrexate, azathioprine, cyclosporine, antihistamines, antidepressants, thalidomide, and phototherapy

A 63‐year‐old man, with a chronic history of severe AD (defined as >10% body surface area involvement and scoring of AD, SCORAD > 20), who had failed multiple systemic therapy. He presented with a history of atopic dermatitis since childhood, which got worse in the past 8 years, increasingly progression with whole body involvement. SCORAD‐66.7% DLQI‐22 (Figure 1).

Previous treatments included topical corticosteroids, tacrolimus ointment, moisturizers, systemic therapy with oral prednisone (1mg/kg/daily) and methotrexate, azathioprine, cyclosporine, antihistamines, antidepressants, thalidomide, and phototherapy, all of them with little or no response. His last attempt was with ustekinumab; however, after 6 months it had no improvement.

**FIGURE 2:**
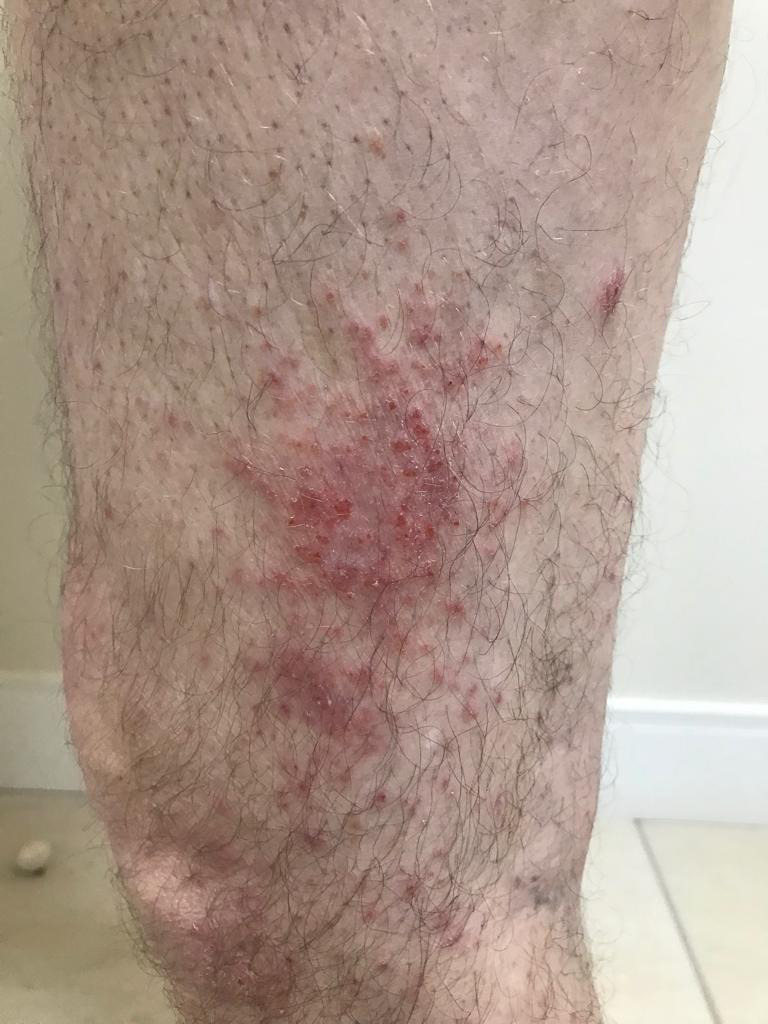
Before treatment with tofacitinib 5 mg/BID

**FIGURE 3:**
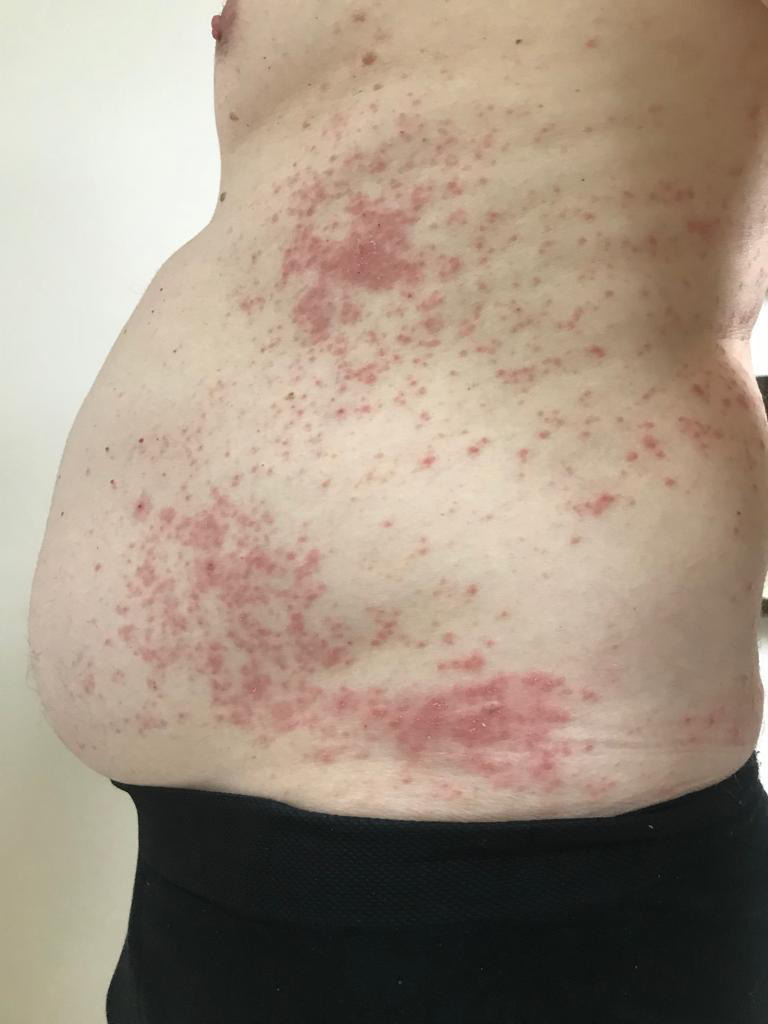
Before treatment with tofacitinib 5 mg/BID

The patient had a family history of inflammatory bowel disease but denied other autoimmune conditions. Complete review of systems was negative. Physical examination revealed reddish, lichenified plaques involving face, trunk, and extremities affecting approximately 25% of total body surface area (Figures 2 and 3). Given the severity of the disease, the limited and inadequate treatment options, with the patient's concern, we decided to treat with tofacitinib citrate by oral route with JAK inhibitors. At this time, dupilumab was not available in Brazil.

**FIGURE 4:**
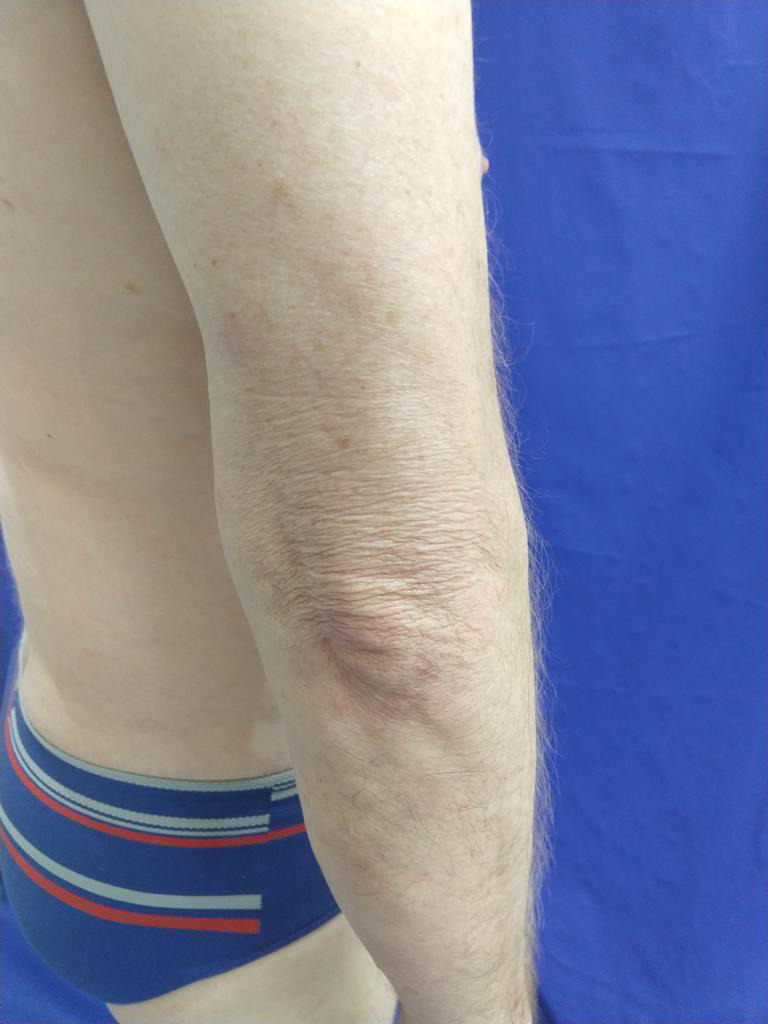
After 3 mo of treatment with tofacitinib citrate 5 mg/BID

**FIGURE 5:**
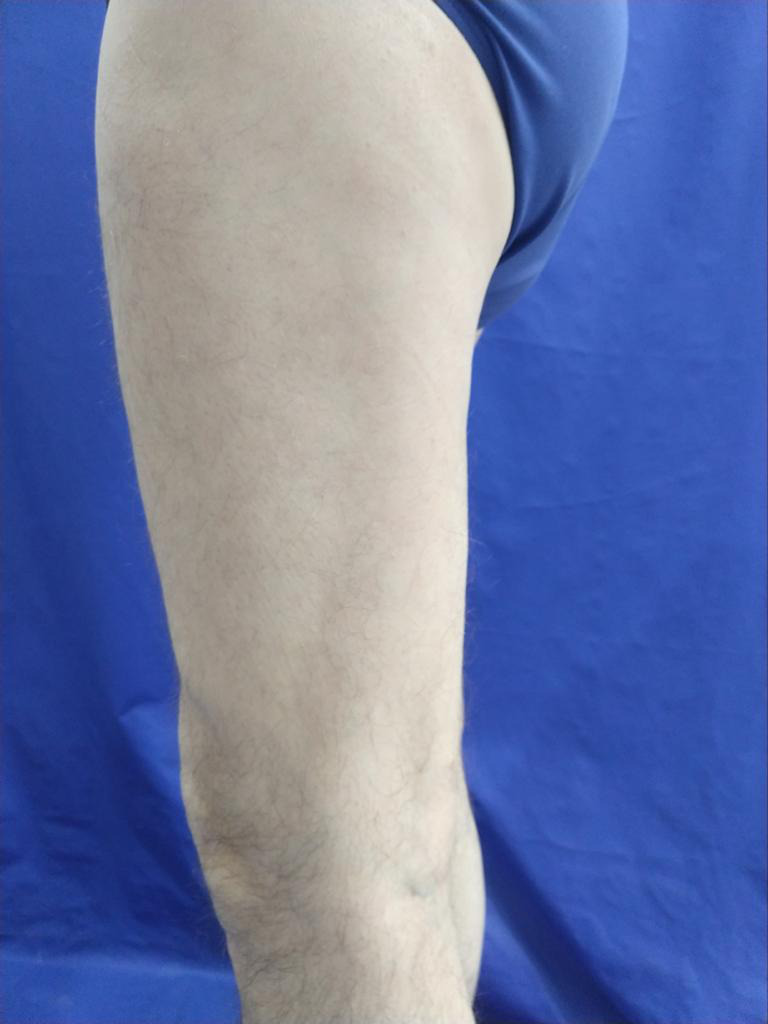
After 3 mo of treatment with tofacitinib citrate 5 mg/BID

Tofacitinib citrate 5mg BID was started. During the first month, he was still on prednisone 20 mg/daily but it was weekly decreased from 20mg to 10mg, then to 5mg until it was totally suspended. During this period, no topical corticosteroids were used, only moisturizers. After three months of treatment, he had nearly complete clearance of face, trunk, upper and lower extremities (Figures 4 and 5). SCORAD‐10.1% DLQI‐3.

At 16 months of follow‐up, he had sustained response on maintenance therapy with tofacitinib 5mg/BID. During treatment, patient developed one episode of uncomplicated herpes simplex infection that was treated with valacyclovir. Laboratory monitoring has not revealed abnormalities.

## 3 DISCUSSION

Atopic dermatitis (AD) is a common, systemic, relapsing, autoimmune inflammatory dermatosis.^1^ AD is characterized by xeroxis, eczematous lesions, marked dryness and redness, persistent, and severe pruritus.^2^ AD is associated with a high burden: sleep disturbance, anxiety and depression and impair quality of life.^3^ The management of atopic dermatitis involves emollients, topical corticosteroids, and calcineurin inhibitors and for refractory disease, immunomodulatory agents, often inadequate for moderate to severe disease. Advances in the understanding of the pathogenesis of AD have allowed new therapeutic approaches.

AD is a predominantly Th2 disease. The key cytokines (IL‐4, IL‐5, IL‐13, IL‐17, IL‐22, IL‐23, IL‐31, IL‐33, and IFN‐y) has a central role in AD pathogenesis,^4^ and Janus kinase (JAK), signal transducer and activator of transcription (STAT) and spleen tyrosine kinase (SYK) pathways are involved in signaling these cytokines. In AD, the use of JAK inhibitors is interesting to block both IL‐4 and IL‐13 in acute phase and interferon y (IFN‐y) in chronic phase.

Tofacitinib citrate, an oral JAk1/3 inhibitor approved for the treatment of arthritis rheumatoid, psoriatic arthritis, and inflammatory bowel disease has already been used to treat other autoimmune disease such as alopecia areata^5^, ^6^ and vitiligo.^7^ Here, we report the successful use of oral tofacitinib for a patient with severe atopic dermatitis refractory to all immunomodulatory drugs previously tried.

## 4 CONCLUSION

Although the use of JAK inhibitors for atopic dermatitis is still off label, oral tofacitinib used for AD has proven to be efficient. In 2015, Levy et al showed efficacy of oral tofacitinib in atopic dermatitis case series.^8^ Then, in 2016, Bissonette et al demonstrated good results with topical tofacitinib in a clinical trial.^9^ And more recently, clinical trials with new JAK ½ inhibitor baricitinib and JAK 1 inhibitors upadacitinib and abrocitinib resulted in improvement in EASI score of patients.^10^, ^11^, ^12^, ^13^ The positive results in case of refractory severe AD suggest that oral tofacitinib can be considered as an therapeutic option. AD is a multifaceted disease so the use of new therapeutic drugs remains an exciting possibility.

## CONFLICT OF INTEREST

None declared.

## AUTHORS’ CONTRIBUTIONS

Sineida Berbert Ferreira: was involved in direct management of the patient. Rachel Berbert Ferreira: reviewed the pertinent literature, the manuscript, and the final draft. Morton Scheinberg: drafted the manuscript and supervised the final draft.

## ETHICAL APPROVAL

The patient agreed with the publication and signed the free and informed consent form.
